# Effect of Melatonin on the *In Vitro* Maturation of Porcine Oocytes, Development of Parthenogenetically Activated Embryos, and Expression of Genes Related to the Oocyte Developmental Capability

**DOI:** 10.3390/ani10020209

**Published:** 2020-01-27

**Authors:** Ling Yang, Qingkai Wang, Maosheng Cui, Qianjun Li, Shuqin Mu, Zimo Zhao

**Affiliations:** 1 College of Life Sciences and Food Engineering, Hebei University of Engineering, Handan 056021, China; yangling@hebu.edu.cn (L.Y.); wangqingkai0814@yeah.net (Q.W.); zhaozm0320@126.com (Z.Z.); 2 Animal Husbandry and Veterinary Research Institute of Tianjin, Tianjin 300412, China; tjlqj@sina.com (Q.L.); mushq@163.com (S.M.)

**Keywords:** melatonin, embryo, oocyte, pig

## Abstract

**Simple Summary:**

Exogenous melatonin has beneficial effects on improving cumulus oophorus expansion; mitochondrial distribution; intracellular level of glutathione; and first polar body extrusion rate of porcine oocytes derived from *in vitro* maturation. Moreover; melatonin supplementation increases relative abundances of *BMP15* and *CAT* mRNA; and decreases intracellular levels of reactive oxygen species; and expression values of *P53* and *BAX* genes; which are related to in vitro development of porcine oocytes.

**Abstract:**

Melatonin treatment can improve quality and in vitro development of porcine oocytes, but the mechanism of improving quality and developmental competence is not fully understood. In this study, porcine cumulus–oocyte complexes were cultured in TCM199 medium with non-treated (control), 10^−5^ M luzindole (melatonin receptor antagonist), 10^−5^ M melatonin, and melatonin + luzindole during in vitro maturation, and parthenogenetically activated (PA) embryos were treated with nothing (control), or 10^−5^ M melatonin. Cumulus oophorus expansion, oocyte survival rate, first polar body extrusion rate, mitochondrial distribution, and intracellular levels of reactive oxygen species (ROS) and glutathione of oocytes, and cleavage rate and blastocyst rate of the PA embryos were assessed. In addition, expression of growth differentiation factor 9 (GDF9), tumor protein p53 (P53), BCL2 associated X protein (BAX), catalase (CAT), and bone morphogenetic protein 15 (BMP15) were analyzed by real-time quantitative PCR. The results revealed that melatonin treatment not only improved the first polar body extrusion rate and cumulus expansion of oocytes via melatonin receptors, but also enhanced the rates of cleavage and blastocyst formation of PA embryos. Additionally, melatonin treatment significantly increased intraooplasmic level of glutathione independently of melatonin receptors. Furthermore, melatonin supplementation not only significantly enhanced mitochondrial distribution and relative abundances of *BMP15* and *CAT* mRNA, but also decreased intracellular level of ROS and relative abundances of *P53* and *BAX* mRNA of the oocytes. In conclusion, melatonin enhanced the quality and in vitro development of porcine oocytes, which may be related to antioxidant and anti-apoptotic mechanisms.

## 1. Introduction

High-efficiency strategies used for in vitro maturation (IVM) of porcine oocytes are indispensable for investigation of female reproductive technologies under in vitro conditions. Nevertheless, quality and developmental competence of oocytes are low under in vitro culture (IVC) systems compared with in vivo produced oocytes and embryos. It is helpful for oocyte IVM; in vitro embryo development under low oxygen concentrations condition in pigs [1]. Reactive oxygen species (ROS) derived from embryo metabolism and culture microenvironment alter the types of most intra- and extracellular molecules, which lead to development blocks and retardation of early embryos [2]. Glutathione, a free radical scavenger, is a major antioxidant that protects cells from ROS damage and maintains cellular redox balance [3]. Melatonin can serve as an antioxidant through upregulating antioxidant enzymes and downregulating prooxidant enzymes [4]. IVM media supplemented with melatonin in an appropriate concentration improves rate of porcine IVM oocytes, and enhances developmental potential of porcine parthenogenetically activated (PA) embryos [5]. Moreover; melatonin addition not only improves quality of bovine cumulus–oocyte complexes (COCs) IVM, but also increases the blastocyst formation rate and quality of bovine PA embryos produced by artificial activation of MII-stage oocytes derived from high-quality COCs [6]. Melatonin addition can increase first polar body extrusion rate and blastocyst rate, and effectively protect the oocytes from heat stress in in vitro porcine oocytes [7]. Medium with 10^−5^ M melatonin enhances blastocyst rates of in vitro PA embryos via melatonin receptors, and luzindole can be used as a melatonin receptor antagonist [8]. Resveratrol and melatonin have synergistic effects on improving oocyte nuclear IVM, total cell numbers of PA blastocysts, and development of porcine nuclear transfer embryos [9].

Mammalian oocyte-secreted factors are implicated in regulation of folliculogenesis and oocyte maturation [10]. Growth differentiation factor 9 (GDF9) and bone morphogenetic protein 15 (BMP15) are essential components of oocyte-secreted factors, and necessary for normal ovarian function [11,12]. Tumor protein p53 (TP53) induces apoptosis and blocks proliferation in various cell types, is implicated in regulation of apoptosis; progesterone secretion; ovarian peptide hormone and prostaglandin secretion in porcine luteinizing ovarian granulosa cells [13]. BCL2 associated X protein (BAX) is an apoptosis regulator, and serves as a signal transduction factor to have proapoptotic roles on granulosa cell apoptosis of porcine atresia follicle [14]. Catalase (CAT) is expressed in oocytes, and required for ROS scavenging, protecting the genome from oxidative damage during meiotic maturation in mouse oocytes [15]. However; the underlying mechanisms of improving quality of porcine oocytes and embryos have not yet been completely understood. 

Effects of melatonin on in vitro maturation of porcine oocytes and intracellular levels of ROS and glutathione of oocytes were analyzed in this study. Cleavage rates and blastocyst rates of the PA embryos were also assessed. Additionally, expression of genes related to oocyte development, including *GDF9*, *BMP15*, *P53*, *BAX*, and *CAT*, were analyzed. The objectives of the present study were to explore the effect of melatonin on in vitro maturation of porcine oocytes, development of parthenogenetically activated embryos, and the relationship of oocyte IVM and developmental capability with expression of genes related to oocyte development in pigs. 

## 2. Materials and Methods

### 2.1. Oocytes Collection and IVM

Experimental procedure was approved by the Animal Care and Use Committee of Animal Husbandry and Veterinary Research Institute of Tianjin, China (AHVRIT-2015049). Porcine ovaries were from a local abattoir, and cumulus–oocyte complexes (COCs) were aspirated from antral follicles (2–8 mm in diameter). All oocytes were selected for IVM with a homogeneous cytoplasm and at least three intact layers of surrounding cumulus cells. Porcine follicle fluid (pFF) was centrifuged and filtered soon after. COCs were rinsed three times using M199 medium (Gibco, Carlsbad, CA, USA) supplemented with follicle-stimulating hormone (FSH; 5 µg/ml; Sigma, St. Louis, MO, USA) and luteinizing hormone (LH; 5 µg/ml; Sigma, St. Louis, MO, USA), 1 IU/ml penicillin and streptomycin (Gibco, Carlsbad, CA, USA), and 20% pFF. The melatonin receptor antagonist group was supplemented with 10^−5^ M luzindole, and the melatonin group was supplemented with 10^−5^ M melatonin. The concentration of melatonin used in this study was based on a study by Shi et al. reporting that melatonin concentration is 10^−3^ to 10^−11^ M during IVM and development of porcine PA embryos [5]. COCs in melatonin + luzindole group were supplemented with 10^−5^ M luzindole and melatonin. COCs in control group were treated with nothing (n ≥ 30 for each group, repeated 3 times). COCs were in the maturation medium at 39 °C, 5% CO_2_, and 100% humidity for 42 h.

### 2.2. Evaluation of Cumulus Expansion, Oocyte Survival Rate, and First Polar Body Extrusion Rate

The degree of cumulus expansion was assessed as described previously [16]. COCs from the four groups were examined at 42 h after IVM culture, and the sum of total COCs scores/total number of COCs was used to calculate degree of cumulus expansion. The score method was as follows: 0, no response; 1, minimum observable response; 2, expansion of outer OCC layers; 3, expansion of all OCC layers except corona radiata; and 4, expansion of all OCC layers [16]. 

The oocyte survival rate was determined as number of survival oocytes/total number of oocytes × 100. The survival oocytes were those that presented zona pellucida and intact plasma membranes, and space between zona pellucida and cell membrane was clear without cytoplasmic leakage or oocyte shrinkage. The oocytes were stained with Hoechst 33342, and assessed the first polar body extrusion rate with a fluorescence microscopy (Nikon Corp., Tokyo, Japan) as described previously [7], and first polar body extrusion rate was the number of oocytes with first polar body/total number of oocytes × 100.

### 2.3. Parthenogenetically Activation

The MII oocytes were washed thrice, and then activated in 0.28 M mannitol supplemented with 0.01% polyvinyl alcohol, 0.1 mM MgCl_2_, and 0.05 mM CaCl_2_ by an electrical pulse of DC 130 V/mm for 80 μs using a BTX Elecro-Cell Manipulator 2001 (BTX, Inc., San Diego, CA, USA). Porcine zygote medium 3 (PZM-3) was used to culture the PA oocytes in an incubator at 39 °C and 5% CO_2_ as described previously [17]. The cleavage rates were checked at 48 h, and blastocyst rate was calculated at 7 days.

### 2.4. Measurement of ROS Level

The ROS levels of oocytes were measured using a reactive oxygen species assay kit (Beyotime Institute of Biotechnology, Haimen, China). Briefly, after cumulus cells and zona pellucida were removed, the matured oocytes from control and melatonin groups (n ≥ 30 for each group, repeated 3 times) were incubated with DCFH-DA (10 mM) at 37 °C for 20 min. The oocytes were checked using a fluorescence microscope (Olympus, Tokyo, Japan) with a filter at 460-nm excitation at the same condition for the two groups in a blind manner. All oocytes were photographed in fluorescence images using a digital camera (Nikon 990, Tokyo, Japan). The fluorescence images were analyzed using the ImageJ by Wayne Rasband from National Institute of Health (Bethesda, MD, USA) to analyze fluorescence intensities of the oocytes compared with that of the control after deducting the background value. The relative fluorescence intensities were the relative ROS levels of the oocytes.

### 2.5. Measurement of Intracellular Glutathione

Glutathione content was determined using a total glutathione assay kit (S0052, Beyotime Institute of Biotechnology, Haimen, China). Briefly, the matured oocytes from four groups (n ≥ 40 for each group, repeated 3 times) were pipetted repeatedly until lysis was completed. Glutathione contents of the oocytes from four groups were measured as described previously [18].

### 2.6. Mitochondrial Distribution Analysis

**Figure 1:**
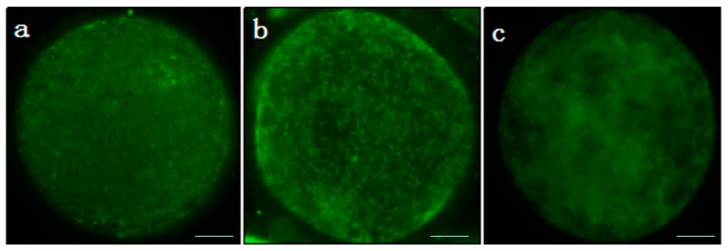
Mitochondrial distribution in porcine oocytes. Oocytes were stained by Mito Tracker Green. (**a**) Green mitochondria distributed evenly within ooplasm; (**b**,**c**) green mitochondria distributed unevenly within ooplasm. Bar = 20 μm.

Oocytes after IVM for 42 h were selected randomly from the control group and melatonin group, and were incubated in pre-warmed maturation medium at 39 °C and 5% CO_2_ for 20 min. A Mito Tracker Green kit (Beyotime Institute of Biotechnology, Haimen, China) was used to label distribution of mitochondria of oocytes at 37 °C for 30 min. The labeled oocytes were checked using a fluorescence microscope (Olympus, BX60, Tokyo, Japan), and all oocytes were photographed using a digital camera (Nikon 990, Tokyo, Japan). There were two main distribution features of mitochondrial distribution patterns in oocyte: one was that labeled mitochondria were distributed evenly throughout ooplasm (homogeneous, Figure 1a), which indicated that mitochondrial distribution was better in oocytes. Others were that the labeled mitochondria were distributed unevenly within ooplasm (heterogeneous; Figure 1b,c) as described previously [19]. The abnormal distribution of mitochondria has negative effects on ATP distribution and embryogenesis [20]. The value of mitochondrial distribution was analyzed in a blind manner, and was determined as the number of oocytes with homogeneous mitochondria/total number of the oocytes × 100.

### 2.7. PCR Assay

Total RNA from the oocytes of the control and melatonin groups was extracted with Trizol reagent (Invitrogen, Carlsbad, CA, USA). Genomic DNA was removed using DNase-I (GeneCopoeia, Rockville, MD, USA). First strand cDNA synthesis kit (GeneCopoeia, Rockville, MD, USA) was used to synthesize cDNA, and qPCR was performed using an All-in-One^TM^ miRNA RT-qPCR detection kit (GeneCopoeia, Rockville, MD, USA) with 7900HT System (Applied Biosystems, Foster City, CA, USA). The primer sequences of *BMP15*, *P53*, *GDF9*, *BAX*, *CAT*, and glyceraldehyde-3-phosphate dehydrogenase (*GAPDH)* were designed and synthesized by Shanghai Sangon Biotech Co., Ltd., China (Table 1). PCR amplification efficiency of each pair of primers was assessed before quantification, and was found to be in an acceptable range (between 0.9 and 1.1). PCR conditions were 40 cycles of 95 °C for 10 s, 55–60 °C (55 °C for *GDF9*; 58 °C for *P53*; 59 °C for *CAT*, 60 °C for *BMP15* and *CAT*) for 20 s, and 72 °C for 25 s. The 2^−ΔΔCt^ analysis method was employed to calculate relative expression levels of *BMP15*, *P53*, *GDF9*, *BAX*, and *CAT* mRNA, with *GAPDH* as control [21].

### 2.8. Statistical Analysis

Each group consisted of three replicates. Chi-squared test was used to analyze the first polar body extrusion rate. Cumulus expansion, in vitro development of PA embryos, intracellular levels of ROS and glutathione, mitochondrial distribution, and expression levels of *BMP15*, *P53*, *GDF9*, *BAX*, and *CAT* were analyzed using oneway ANOVA with Duncan’s test for post hoc analysis in SAS version 8 (SAS Institute Inc., Cary, NC, USA). Data were expressed as mean ± standard deviation. *p* < 0.05 was deemed statistically significant.

## 3. Results

### 3.1. Cumulus Expansion, Survival and First Polar Body Extrusion Rates of Oocytes, and in Vitro Development of PA Embryos in Pigs

The results showed that degree of cumulus expansion of COCs and first polar body extrusion rate of the oocytes from the melatonin group were the highest among the four groups (*p* < 0.05), but melatonin addition had no effects on the melatonin + receptor antagonist group (*p* > 0.05; Table 2). Furthermore, melatonin treatment did not affect survival rate of oocytes (*p* > 0.05; Table 2) or the first polar body extrusion rate of the oocytes from melatonin + receptor antagonist group.

It was shown in Table 2 that cleavage rate and blastocyst rate of the PA embryos from the melatonin group were the highest among the four groups (*p* < 0.05), but there was no significant improvement in the melatonin + receptor antagonist group (*p* > 0.05).

### 3.2. Intracellular Levels of ROS and Glutathione; and Mitochondrial Distribution in the Oocytes

**Figure 2:**
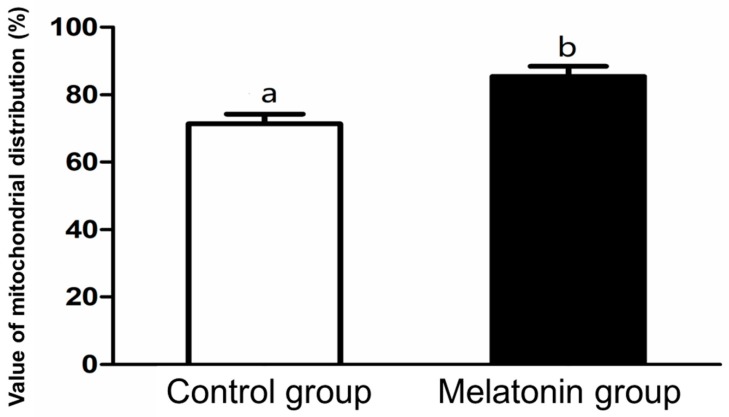
Effect of melatonin addition on the value of mitochondrial distribution in porcine oocytes after in vitro maturation. High value indicates that mitochondrial distribution in oocyte is more homogeneous. Different superscript letters within the different column indicate significantly different (*p <* 0.05).

**Figure 3:**
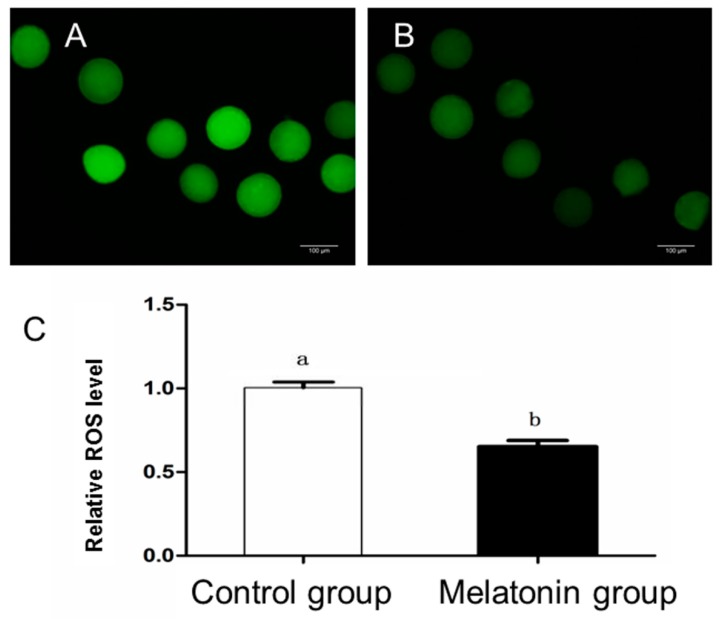
Effect of melatonin on reactive oxygen species (ROS) level of oocytes after in vitro maturation. (**A**) Representative image of ROS level in the control group. (**B**) Representative image of ROS level in the melatonin group. (**C**) The relative ROS levels in the control group and melatonin group. Different letters in the different column represent significant difference (*p* < 0.05). Bar = 100.

As shown in Table 3, glutathione levels in the oocytes from the melatonin group and the melatonin + receptor antagonist group were significantly higher than that from the groups with no melatonin supplementation (*p* < 0.05). Furthermore, the value of mitochondrial distribution of the oocytes from the melatonin group was significantly high comparing with that from the control group (*p* < 0.05; Figure 2), but intracellular ROS levels in the oocytes from the melatonin group was significantly low compared with that from the control group (*p* < 0.05; Figure 3).

### 3.3. Expression of Genes Related to the Oocyte Developmental Capability

**Figure 4:**
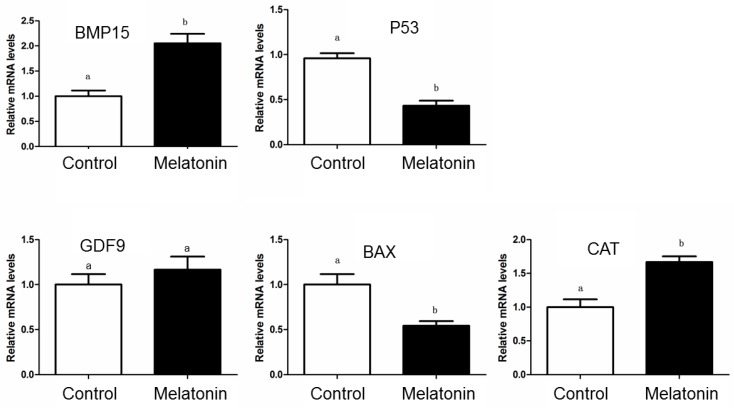
Effect of melatonin on expression of *BMP15*, *P53*, *GDF9*, *BAX*, and *CAT* mRNA in the porcine oocytes after in vitro maturation. Different letters in the different color column represent significant difference (*p* < 0.05).

The RT-qPCR results showed that relative abundances of *BMP15* and *CAT* mRNA in the oocytes from melatonin group were significantly high comparing with that from control group (Figure 4; *p* < 0.05), but melatonin treatment had negative effects on relative abundances of *P53* and *BAX* mRNA in the oocytes (*p* < 0.05). However, melatonin treatment did not affect relative abundance of *GDF9* mRNA in the porcine oocytes (*p* > 0.05).

## 4. Discussion

Our data indicated that melatonin treatment affected cumulus oophorus expansion and first polar body extrusion rate of the oocytes during IVM, but melatonin **+** receptor antagonist had no significant effects. Normal cumulus expansion is necessary for nuclear and cytoplasmic maturation of oocytes in pigs [22]. Melatonin has a wide range of roles in physio-pathological functions, and is partly mediated by melatonin receptors in animals [23]. Melatonin treatment improves cumulus oophorus expansion of porcine COCs during IVM [24], and also increases the first polar body extrusion rate of the oocytes disrupted by heat stress and mono-(2-ethylhexyl) phthalate exposed in the porcine [7,25]. Melatonin participates in modulating functions of granulosa cell through melatonin receptor 2 (MT2), and also promotes cumulus expansion of COCs via MT2 in pigs [26]. Therefore, melatonin treatment can enhance cumulus oophorus expansion and the first polar body extrusion rate of oocytes through melatonin receptors, which is beneficial for nuclear and cytoplasmic maturation of porcine oocytes during IVM.

Melatonin treatment was beneficial for increasing the cleavage rate and blastocyst rate of PA embryos in this study, and treatment with melatonin + receptor antagonist had no effect on cleavage rate or blastocyst rate in pigs. Culture media supplemented with melatonin are helpful for improving developmental capability and quality of in vitro fertilized embryos in the porcine [27], and melatonin treatment can improve developmental potential of in vitro PA embryos in pigs [28]. Melatonin treatment could protect in vitro oocytes and PA embryos from toxicity (impaired development rate and blastocyst quality) induced by aflatoxin B1 in the porcine [29]. The MT1 receptor is involved in improving development of in vitro embryo by melatonin treatment in cattle [30]. Melatonin can remarkably alleviate oxidative stress, and markedly promote in vitro embryonic development from the oocytes aged for 24 h in pigs [31]. Therefore, it is indicated that melatonin treatment is helpful for development of in vitro PA embryos, which is via melatonin receptors.

It was found in this study that culture media supplemented with melatonin enhanced intracellular glutathione level, decreased ROS level of the porcine oocytes, and receptor antagonist did not affect the intracellular level of glutathione during IVM. Embryos can change environmental conditions, including level of oxygen, which induces a rise of ROS. ROS can modify biological molecules, and induce abnormal development or even embryonic lethality [32]. Melatonin achieves its detoxification of reactive oxygen through inducing antioxidant enzymes [33]. Benzo(a)pyrene leads to oocyte meiotic failure, which can be recovered by melatonin supplementation through repressing ROS level in the porcine [34]. Melatonin combined with prolonged IVM enhances development of poor-quality oocytes through decreasing ROS generation in pigs [35]. The combination of GSH with L-cysteine decreases ROS production, which can be used to enhance blastocyst quality in IVC systems in pigs [36]. Melatonin treatment improves IVM of oocyte under heat stress via increasing GSH level, reducing ROS level in porcine oocytes [37], and melatonin treatment enhances oocyte quality during IVM through upregulating intracellular GSH and ATP, and expression of antioxidant genes in cattle [38]. Therefore, melatonin treatment is beneficial for oocyte IVM through upregulating intracellular glutathione and downregulating intracellular ROS in oocytes, which is not via melatonin receptors in porcine oocytes.

Our results showed that the value of mitochondrial distribution of the oocytes was significantly higher in the melatonin group after IVM, which indicated that melatonin treatment could increase the percentage of oocytes with homogeneous mitochondria. Mitochondria are important cell organelles, and implicated in many cellular activities including apoptosis [39]. Mitochondrium is essential for oocyte functions, fertilization, and development competence, and are also important indicators of oocyte quality [40]. Mitochondria in oocytes and cumulus cells play vital roles in oocyte developmental competence, and can serve as a marker of developmental competence in porcine oocytes and cumulus cells [41]. Paraquat exposure causes abnormal distribution patterns of mitochondria in in vitro bovine oocytes, and melatonin treatment improves mitochondrial functionality of the oocytes exposed to paraquat during IVM in the bovine [42]. IVM medium supplemented with melatonin can alter mitochondrial distribution patterns of oocytes, but do not affect mitochondrial activity of bovine oocytes [43], and defective mitochondrion integrity induced by benzo(a)pyrene can be recovered by melatonin treatment via inhibiting ROS level in the porcine oocytes [34]. Therefore, melatonin can enhance mitochondrial distribution of oocytes during IVM, which is helpful for functions and development competence of in vitro oocytes.

This study indicated that melatonin treatment improved expression of *CAT* mRNA in the oocytes during IVM. CAT plays key roles in neutralizing harmful hydrogen peroxide from various sources [44]. *CAT* mRNA expression in ovarian granulosa cells (GCs) decreases following β-zearalenol and HT-2 treatment, but upregulates in bovine melatonin-treated GCs [45]. Melatonin treatment improves frozen–thawed semen quality, and increases blastocyst development rate and *CAT* transcript abundance of the bovine in vitro-produced embryos originating from melatonin-treated spermatozoa [46]. Melatonin treatment improves CAT activity in ovaries, and protects against clomiphene citrate-induced egg apoptosis in rats [47]. Melatonin treatment improves cytoplasmic maturation of in vitro oocytes, and increases intracellular ROS level and expression level of *CAT* in cattle [38]. Therefore, upregulation of glutathione and downregulation of ROS induced by melatonin may be via enhancing CAT expression in porcine oocytes during IVM.

This study demonstrated that melatonin treatment induced expression of *BMP15* mRNA, but had no significant effects on *GDF9* mRNA expression in the oocytes during IVM. BMP15 and GDF9 are members of transforming growth factor-beta superfamily [48]. BMP15 participates in regulating follicle growth and oocyte developmental competence [49]. GDF9 and BMP15 exert integral actions on oocyte quality and fetal growth [50], and are implicated in oocyte development, fertilization, and embryonic competence in heterodimers or homodimers through autocrine and paracrine manners in women [51]. In humans, there is a positive relation between expression values of *GDF9* and *BMP15* mRNA in cumulus granulosa cells and oocyte maturation, fertilization, and embryo quality [52]. Oocytes and follicular cells express *GDF9* mRNA during oocyte-cumulus complex IVM in pigs [16]. GDF9 and BMP15 are limited to oocyte cytoplasm, and GDF9 and BMP15 addition improves expression of genes related with oocyte maturation and cumulus expansion in porcine oocytes during IVM [53]. Melatonin treatment moderates reduction of relative values of *BMP15* and *GDF9* mRNA caused by oocyte denudation during IVM in the bovine [6], and also upregulates expression of *BMP15* and *GDF9* in inferior oocytes, which are beneficial for oocyte maturation and embryo development in cattle [54]. Melatonin supplementation enhances expression of *GDF9* gene via melatonin membrane receptors in oocytes, which are beneficial for bovine oocyte IVM [55]. There is a low expression of value *BMP15* mRNA in immature oocytes, and *BMP15* mRNA is upregulated at 18 h of IVM in porcine oocytes. However, *GDF9* mRNA is upregulated in porcine immature oocytes, but downregulated during IVM [56]. Therefore, it may be mainly through upregulating BMP15, but not GDF9, via melatonin membrane receptors that melatonin supplementation enhances oocyte IVM and development competence in the porcine.

Our results found that melatonin supplementation inhibited expression of *P53* and *BAX* mRNA in the oocytes during IVM. P53 induces apoptosis through direct interactions with both chromatin and regulators of transcription in multicellular organisms [57], and cytoplasmic P53 can lead to transcription-independent neural precursor cell apoptosis through interaction with activated BAX [58]. Melatonin treatment improves development of bovine nuclear transfer embryos, and inhibits *P53* and *BAX* expression [59]. Vitrification solution or/and IVM solution supplemented with melatonin can decrease ROS level and *BAX* mRNA expression, which improve developmental ability of oocytes in cattle [60]. Melatonin delivery by nanocapsules is more effective than melatonin treatment without nanocapsules in improving cleavage rate and blastocyst rate and downregulating expression of *BAX* gene during oocyte IVM in cattle [61]. Exogenous melatonin improves development of cloned embryos via suppressing the P53-mediated apoptotic pathway, through directly scavenging free radicals in pigs [62]. Melatonin treatment reduces expression of *BAX* and *P53* mRNA through activating MT2 in granulosa cells of pigs [63]. Therefore, downregulation of *BAX* and *P53* is involved in melatonin-induced improvement of quality of IVM oocytes and development competence of in vitro PA embryos in pigs.

## 5. Conclusions

Exogenous melatonin can improve cumulus oophorus expansion, mitochondrial distribution, intracellular level of glutathione, and first polar body extrusion rate of IVM-derived porcine oocytes. Moreover, melatonin treatment increases the relative abundances of *BMP15* and *CAT* mRNA in IVM oocytes. Simultaneously, melatonin treatment decreases both the intracellular level of ROS and expression levels of *P53* and *BAX* mRNA in IVM-derived oocytes, which turn out to improve quality and outcome of IVM oocytes and development competence of PA embryos in pigs. Furthermore, the beneficial effects of melatonin on cumulus expansion, first polar body extrusion rates of oocytes, and in vitro development of PA embryos are dependent on melatonin receptors, but the intracellular level of glutathione is independent of melatonin receptors. All in all, our finding may provide a base for improving quality of IVM oocytes and IVC embryoes. Further experiments may be needed to determine the relationship of exogenous melatonin to expression of BMP15, CAT, P53, and BAX in protein levels.

## Figures and Tables

**Table 1:** Primer sequences.

Gene	Primer Sequence	Product Size	GenBank Accession No.
*GAPDH*	F: TCAAATGGGGTGATGCTGGT R: GCAGAAGGGGCAGAGATGAT	124 bp	XM_021091114
*BMP15*	F: AGCACAACCAGTCACTTTCCT R: CCCCTTGTGATTCCAGAGCT	123 bp	NM_001005155
*P53*	F: AAGACCTACCCTGGCAGCTA R: ACAGCTTATTGAGGGCAGGG	100 bp	NM_213824
*GDF9*	F:AGCCAGACTCCAGAGCTTTG R: TGAAGAGCCGGACAGTGTTG	114 bp	NM_001001909.1
*BAX*	F: GCTTCAGGGTTTCATCCAGGA R: CCAGTTCATCTCCAATGCGC	134 bp	XM_003127290
*CAT*	F: ACGTTGGAAAGAGGACACCC R: TCCAACGAGATCCCAATTACCA	137 bp	NM_214301

*GAPDH: Glyceraldehyde-3-phosphate dehydrogenase; BMP15: Bone morphogenetic protein 15; P53: Tumor protein p53; GDF9: Growth differentiation factor 9; BAX: BCL2 associated X protein; CAT: Catalase.*

**Table 2:** Effects of melatonin and melatonin receptor inhibitor (Luzindole) on cumulus expansion, survival and first polar body extrusion rates of oocytes, and in vitro development of PA embryos in pigs.

In Vitro Maturation of Oocyte and Development of Embryo	Control	Melatonin	Melatonin + Luzindole	Luzindole
Degree of cumulus expansion (n)	2.74 ± 0.07 ^a^ (421)	2.86 ± 0.08 ^b^ (385)	2.76 ± 0.08 ^a^ (431)	2.72 ± 0.06 ^a^ (431)
Survival rate % (n)	94.23 ± 0.86 ^a^ (408/433)	95.67 ± 0.19 ^a^ (420/439)	93.96 ± 0.79 ^a^ (420/447)	94.91 ± 1.62 ^a^ (410/432)
First polar body extrusion rate %	79.66 ± 1.89 ^a^ (325/408)	85.71 ± 2.26 ^b^ (360/420)	80.48 ± 2.50 ^a^ (338/420)	80.49 ± 1.10 ^a^ (330/410)
Cleavage rate %	77.48 ± 2.05 ^a^ (117/151)	85.63 ± 2.50 ^b^ (142/167)	79.87 ± 1.86 ^a^ (119/149)	76.54 ± 3.26 ^a^ (124/162)
Blastocyst rate %	29.91 ± 1.75 ^a^ (35/151)	35.92 ± 3.90 ^b^ (51/142)	30.25 ± 1.82 ^a^ (36/119)	29.03 ± 2.07 ^a^ (36/124)

Note: ^a,b^ Significantly different (*p* < 0.05) was indicated by different letters within the same row.

**Table 3:** Glutathione concentration in porcine oocytes.

Group	Number of Oocytes	Replicates	Glutathione Concentration (Pmol/Oocyte)
Control	40	3	5.10 ± 0.13 ^a^
Melatonin antagonist	40	3	5.52 ± 0.35 ^a^
Melatonin	40	3	6.22 ± 0.21 ^b^
Melatonin + receptor antagonist	40	3	6.21 ± 0.45 ^b^

Note: ^a,b^ Significantly different (*p* < 0.05) was indicated by different letters within the same column.
